# Postdural Puncture Headache

**DOI:** 10.1155/2010/102967

**Published:** 2010-08-11

**Authors:** Ahmed Ghaleb

**Affiliations:** Department of Anesthesiology, University of Arkansas for Medical Sciences, 4301 West Markham, Slot 515, Little Rock, AR 72205, USA

## Abstract

Postdural puncture headache (PDPH) has been a problem for patients, following dural puncture, since August Bier reported the first case in 1898. His paper discussed the pathophysiology of low-pressure headache resulting from leakage of cerebrospinal fluid (CSF) from the subarachnoid to the epidural space. Clinical and laboratory research over the last 30 years has shown that use of small-gauge needles, particularly of the pencil-point design, is associated with a lower risk of PDPH than traditional cutting point needle tips (Quincke-point needle). A careful history can rule out other causes of headache. A postural component of headache is the sine qua non of PDPH. In high-risk patients , for example, age < 50 years, postpartum, large-gauge needle puncture, epidural blood patch should be performed within 24–48 h of dural puncture. The optimum volume of blood has been shown to be 12–20 mL for adult patients. Complications of AEBP are rare.

## 1. Introduction

PDPH is an important iatrogenic cause of patient morbidity in modern day anesthesia and pain management practice after attempted epidural block. The incidence of dural puncture, in the literature, ranges from 0.16% to 1.3% in experienced hands [1]. Postdural puncture headache develops in 16%–86% after attempted epidural block with large bore needles [2].

Any breach in the dura may result in PDPH. A breach can be either iatrogenic or spontaneous. Performing an epidural, spinal, or a diagnostic myelogram can produce the very distinct PDPH. It usually occurs within 48 hours postprocedure. Spontaneous CSF leaks leading to headache are usually seen in the cervical-thoracic region and are also associated with comorbidities like Marfan's syndrome, neurofibromatosis, connective tissue disorders, and Ehler's Danlos [3].

According to the International Headache Society, the criteria for PDPH [4] include a headache that develops less than seven days after a spinal puncture, occurs or worsens less than fifteen minutes after assuming the upright position, and improves less than thirty minutes in the recumbent position with at least one of the following (neck stiffness, tinnitus, hypacusia, photophobia, and nausea). The headache should disappear within fourteen days after a spinal puncture; if it persists, it is called a CSF fistula headache.

## 2. History

The history of spinal anesthesia can be traced back to the late 1800s when Wynter and Quincke aspirated cerebrospinal from patients with tuberculous meningitis in an attempt to lower intracranial pressure [5]. Shortly thereafter, John Corning attempted to use spinal cocaine injection for the treatment of habitual masturbation. Whether it cured the ailment it was intended for is unknown. August Bier performed spinals on himself and eight other subjects using 10–15 mg of cocaine. Four of the nine people, including Professor Bier, developed PDPH [6].

## 3. Anatomy and Pathophysiology

Anatomically, the spinal dura mater extends from the foramen magnum to the second segment of the sacrum. It consists of dense connective tissue matrix of collagen and elastic fibers. The average adult produces about 500 mL of CSF per day, or 21 mL per hour (0.3 mL/kg/hr), with 90% coming from the choroid plexus, and 10% from the brain substance itself. A total of about 150 mL of CSF circulates at any one time and is absorbed by arachnoid villi. The cause of PDPH is not entirely certain. The best explanation is that low CSF pressure results from CSF leakage through a dural and arachnoid tear; a leakage that exceeds the rate of CSF production [4]. As little as 10% loss of CSF volume can cause an orthostatic headache. There are two basic theoretical mechanisms to explain PDPH. One is reflex vasodilatation of the meningeal vessels due to the lowered CSF pressure. The other is the traction on the pain sensitive intracranial structures in the upright position. The traction on the upper cervical nerves like C1, C2, C3 causes the pain in the neck and shoulders. Traction on the fifth cranial nerve causes the frontal headache. Pain in the occipital region is due to the traction of the ninth and tenth cranial nerves.

## 4. Needle Size and Incidence of PDPH

The incidence of PDPH is directly related to the needle diameter that pierces the dura mater [7]. Although smaller diameter needle punctures used for subarachnoid block decrease the risk of PDPH, these needles are technically difficult to use and are associated with a lower success rate of spinal anesthesia [8], especially in inexperienced hands. This is due to failure in recognizing dural puncture secondary to slow flow through a small needle, leading to multiple and repeated puncture attempts. The incidence of PDPH with the 25-gauge Whitacre (non cutting) needle is less than with the 27-gauge Quincke (cutting) needle [7]. Morbidity associated with lumbar puncture can be decreased by the proper selection of an appropriate needle gauge and needle tip configuration [9, 10].

## 5. Direction of Bevel

Spinal needles are designed as cutting bevels, as in the Quincke-type, or pencil point, as in the Whitacre-type spinal needle. Dural fibers were once believed to run longitudinally [11]; however microscopic dissection of the dura mater from cadavers revealed that dural fibers do not run longitudinally or in parallel fashion. The dura is a laminated structure built up from well-defined layers oriented concentrically around the medulla spinalis [12]. Orienting the bevel of a cutting needle probably needs further consideration before making absolute, blanket statements regarding the etiology of dural puncture leaks. The use of a paramedian approach to the subarachnoid space has been suggested as a means of reducing PDPH particularly when using cutting needles [13].

Electron microscopy has shown that pencil point needles are more traumatic to the dura than the cut bevel needles. It is postulated that a pencil point needle produces an irregular tear in the dura and the subsequent inflammatory reaction reduces CSF leakage more effectively than the clean U-shaped puncture seen with a cutting-bevel needle, which decreases the risk of PDPH [14].

## 6. Dura Mater and Response to Trauma

After perforation of the dura, there will be leakage of CSF. In neurosurgical experience even minor perforations need to be closed, either directly or through the application of synthetic or biological dural graft material. Failure to close the dural perforation may lead to adhesions, continuing CSF leak, and the risk of infection. It was thought that the closure was facilitated through fibroblastic proliferation from the cut edge of the dura. Work published in 1959 [15] dismissed the notion that the fibroblastic proliferation arose from the cut edge of the dura. This study maintained that the dural repair was facilitated by fibroblastic proliferation from surrounding tissue and blood clot. The study also noted that dural repair was promoted by damage to the pia-arachnoid, the underlying brain, and the presence of blood clot. It is therefore possible that a spinal needle carefully placed in the subarachnoid space does not promote dural healing; as trauma to adjacent tissue is minimal. Indeed, the observation that blood promotes dural healing agrees with Gormley's original observation that bloody taps were less likely to lead to a postdural puncture headache as a consequence of a persistent CSF leak [16].

## 7. Symptoms

PDPH typically manifests as a postural, frontal, frontotemporal, or occipital headache, worsened by ambulation and improved by assuming the decubitus position, occurring within 48 hours after dural puncture. The accompanying symptoms are usually nausea, vomiting, and neck stiffness [1, 17]. Atypical symptoms after accidental dural puncture have been infrequently described.

 Other nonspecific symptoms may occur such as nausea, vomiting, and ocular complaints such as photophobia and diplopia, and auditory complaints like tinnitus and hyperacusis. The first case of diplopia after dural puncture was reported by Quincke more than 100 years ago [17]. Diplopia or extraocular muscle paralysis (EOMP) after dural puncture has been reported occasionally, primarily in the neurology and ophthalmology literature. Because there seems to be a window period before diplopia manifests after dural puncture, the patient and physician may not always believe that the symptom is secondary to dural puncture, particularly when it occurs after resolution of PDPH. Diplopia usually occurs 4–10 days after dural puncture but can manifest as late as 3 weeks. Full recovery can generally be expected in 2 weeks to 8 months, although permanent cases have rarely been reported.

## 8. Factors Influencing Incidence

Women, particularly during pregnancy and especially after vaginal delivery, are considered at increased risk for PDPH. The incidence of PDPH is highest between 18 and 30 years of age and declines in children younger than 13 years and adults older than 60 years. The incidence is greater in patients with lower body mass index [2]. Women who are obese or morbidly obese may actually have a decreased incidence of PDPH. This may be because the increase in intra-abdominal pressure may act as an abdominal binder helping to seal the defect in the dura and decreasing the loss of CSF. Younger women may be at a greater risk because of increased dural fiber elasticity that maintains a patent dural defect compared to a less elastic dura in older patients [4]. Patients with a headache before lumbar puncture and a prior history of PDPH are also at increased risk. There is no known relationship between the diagnosis of migraine headaches and increased incidence of PDPH after regional anesthesia [18]. There may be some correlation between history of motion sickness and PDPH. Another important factor is the experience of the person doing the procedure leading to the puncture of the dura. Continuous spinal reduced the incidence of PDPH when compared to single shot spinal, at least according to one study mentioned in [19].

## 9. Differential Diagnosis

A comprehensive history and physical exam must be carried out before making the diagnosis of PDPH. Spinal abscess, spinal hematoma, septic or aseptic meningitis, intracranial mass lesion, cerebral aneurysm, cerebral edema, myofascial syndrome, arachnoiditis caused by intrathecal steroids, transient neurologic syndrome or related symptoms, unspecific postdural puncture lumbalgia, neural toxicity of the drugs, and anterior spinal artery syndrome should all be ruled out [8, 20, 21]. Additional tests such as magnetic resonance imaging could be performed in cases with atypical postdural puncture symptoms, to exclude the possibility of developing serious complications.

Few cases of atypical postdural puncture symptoms have been reported in the literature. Keener cited interscapular pain as a “related musculoskeletal symptom,” however no instances of upper back pain are cited among the 75 cases of PDPH reported by the author [15]. McGrady and Freshwater reported a case of posterior neck pain without headache after spinal anesthesia [22]. Errando et al. reported a case of arm pain with dysesthesia after an unintended dural puncture and explained it as irritation of the C5 and C6 nerve roots caused by central traction [21].

## 10. Treatment

### 10.1. Conservative/Symptomatic Therapy

The treating clinician must provide emotional support and reassurance to patients with PDPH. Bed rest has been advocated in cases of dural puncture by some clinicians. However, a recent meta-analysis failed to show that bed rest after dural puncture was better than immediate mobilization in reducing the incidence of PDPH [23]. Bed rest can be associated with a higher incidence of PDPH in particular patient groups [24]. Bed rest may postpone the occurrence of the headache but does not prevent it.

### 10.2. Pharmacotherapy

#### 10.2.1. Oral and Intravenous Medications

Oral hydration remains a popular therapy for PDPH, but there is no evidence that vigorous hydration has any therapeutic benefit, or that it encourages an increased production of cerebrospinal fluid. However, no patient with PDPH should be allowed to become dehydrated. 

The efficacy of oral caffeine for the treatment of PDPH was evaluated in 40 postpartum patients [25]. A single oral dose was demonstrated to be safe, effective and should be considered in the early treatment of mild PDPH. Caffeine sodium benzoate, as an intravenous bolus or an infusion, can be used to treat PDPH. Caffeine was 75% to 80% effective in the initial treatment of PDPH; however, follow-up 48 hours later revealed that all patients had a return of their headache [26]. Methylxanthines may block cerebral adenosine receptors, which lead to vasoconstriction of dilated cerebral blood vessels, but the effect is transient. 

Cosyntropin, a synthetic form of adrenocorticotropic hormone, has been used in the treatment of refractory PDPH. Adrenocorticotropic hormone is believed to work by stimulating the adrenal gland to increase CSF production and *β*-endorphin output. Caution should be used in patients with diabetes [27]. 

The serotonin type 1-d receptor agonist (Sumatriptan) is effective in the treatment of PDPH, with complete resolution of symptoms [28, 29]. The drug is expensive, and side effects include pain at the site of injection and chest tightness. Caution must be used in treating patients with ischemic heart disease using sumatriptan [28, 29]. Controlled trials are needed to further evaluate the use of sumatriptan for PDPH.

A trend away from conservative management to blood patch has appeared in the recent years. This is based on the relative ineffectiveness of the conservative treatment. For example, over 80% of postpartum patients who were conservatively treated will still have headache by one week.

#### 10.2.2. Epidural Injections

AEBP has become the “gold standard” in the treatment of PDPH. As there is some risk of infection when injecting blood into the epidural space, we will discuss the efficacy of some other aqueous agents that have been injected into the epidural space to treat PDPH. Prior to considering the use of epidural injections of blood or other substances to relieve the symptoms of PDPH, there needs to be a clearly negative history of sepsis and coagulopathy. HIV infection is not considered to be a contraindication to AEBP.

Dextran and 0.9% NaCl (saline) injections into the epidural space transiently increase pressure in the epidural space, which subsequently decreases the leakage of CSF and restores subarachnoid pressure [30–32]. Not only the success rate moderate, but also anaphylaxis has been reported following the use of dextran for this purpose [31]. Epidural patching with nonblood substances, for example, saline or colloid, is ineffective for prolonged relief [33], although other substances such as fibrin glue have been used [34].

#### 10.2.3. Autologous Epidural Blood Patch (AEBP)

The AEBP was first described by Gormley in 1960 for use in PDPH and was later popularized by Crul et al. [35] and DiGiovanni and Dunbar [36]. The suspected mechanism of action of AEBP is tamponade of the dural leakage while simultaneously raising the subarachnoid pressure. Elevation of subarachnoid and epidural pressures remains so only for about 20 minutes [37]. MRI evidence confirms a mass effect after injection of epidural blood, with gradual resolution over about 7 hours. Unlike saline, dextran, or other fluids, blood is not removed quickly from the epidural space [38], and it potentially exerts a tamponade effect for much longer periods of time. The autologous blood is thought to form a fibrin clot over the dural rent, allowing CSF volume and hence pressure to normalize as new CSF is generated [39]. 

Abouleish et al. summarized 524 cases of AEBP reported by 11 centers [40]. Persistent symptomatic relief of PDPH following epidural blood patch was >95%, particularly when using volumes of blood >15 mL. In this paper, using volumes of blood greater than 20 mL offered no advantages, as it is known that 20 mL spreads about 9-10 spinal segments when administered to patients in the sitting position [10].

Some studies have demonstrated lower success rates, with only 61%–75% of patients demonstrating sustained benefit. These lower success rates may reflect dural puncture occurring with large-bore epidural needles versus smaller-gauge spinal needles [30, 41, 42]. In obstetrical studies, the success rate of epidural blood patch for PDPH is lower because the dural hole made by 18 gauge Tuohy needles results in a large leakage of CSF, necessitating a second blood patch in as many as 29% of patients [30, 42].

#### 10.2.4. The Technique of AEBP

The procedure is performed only after a careful history to exclude other causes of headache. While some authors have recommend the administration of prophylactic antibiotics for the procedure, they are generally not used. Rarely, if fluoroscopy is utilized, the prone position may be selected. The preferred interspace for injection is the one below the previous injection site, because blood preferentially rises cephalad following its injection into the lumbar epidural space [38, 43]. Usually, at least 20 mL of blood are aseptically withdrawn. In children, 0.2-0.3 mL/kg of blood is needed. Phlebotomy should be attempted after first identifying the epidural space to avoid clotting. The blood is carefully, and aseptically, transferred to the anesthesiologist, who injects it slowly through the epidural needle until one of the following endpoints occurs: (a) complaint of back pain, neck pain, or radicular pain in the leg or worsening headache during the performance of the epidural injection, or (b) once at least 20 mL have been successfully injected without complaint by the patient. The patient is advised to avoid straining, bending, or heavy lifting for 2-3 days to allow the dural hole to heal. 

In regard to the optimum volume of autologous blood to be injected epidurally, Abouleish et al. found that using 10 mL standard in all patients was equivalent of 10–15 mL variably administered based upon height [40]. Others have advocated more generous volumes. Crawford found that 20 mL was associated with 96% success versus 70% success using 6–15 mL [44]. The ideal time to perform an epidural blood patch is still controversial as some authors believe that AEBP performed within 24 hours has a low success rate; others believe that the ideal time is within 24 hour of puncture [45]. Treatment failure after blood patch may reflect continued transdural leak [46]; in this case, the blood patch should be repeated while keeping the patient flat for 24 hours afterwards to reduce the flow of CSF through the dural rent.

Complications following AEBP include the following: backache (35%), neck pain (0.9%), and transient temperature elevations (5%) lasting 24–48 hours. Bleeding, infection, repeat dural puncture, and arachnoiditis from blood injected into the subarachnoid space have been reported. There have been at least two cases of facial nerve paralysis reported following autologous blood patch, both of which resolved spontaneously. Significant complications following large-volume epidural blood patches for postdural puncture headache were reported. A 39-year-old woman developed a spinal subdural hematoma causing both lumbar back and radicular pain following a single LEBP using 58 mL of blood. The second case was a 33-year-old woman who received three LEBPs over a 4-day period totaling 165 mL of blood. She developed arachnoiditis and chronic sacral radiculopathy with resolution 4 months later. Lowe and McCullough suggested that the etiology is ischemia of the 7th nerve resulting from decreased blood supply after an increase in intracranial pressure due to the injection of blood in the epidural space [47]. There has also been at least one case of intractable dizziness, vertigo, tinnitus, and ataxia [48, 49]. Blood patch has occasionally been associated with vasovagal syncope [50].

#### 10.2.5. Prophylactic Blood Patch

Some have suggested that blood patch be performed as a prophylactic measure (i.e., prior to the development of a headache) in cases of unintended dural puncture occurring after the insertion of 17-18 gauge epidural needle into the subarachnoid space, particularly when there has been loss of considerable quantities of CSF. To date, there have been no large, prospective studies to advocate this practice, although some anesthesia practitioners still use it. Existing studies regarding prophylactic epidural blood patch are limited by small patient numbers [51, 52]. Also, there have been limitations suggested by the small volumes of blood injected in some reviews [53]. If one chooses to perform prophylactic AEBP, some caveats are in order; one should avoid prophylactic AEBP immediately following local anesthetic (LA) epidural top-off dose administration, because the resultant high epidural pressure has resulted in at least one case of total spinal block [54]. Also, the presence of LA in the epidural space may theoretically interfere with subsequent blood clot formation [55]. Some have strongly advocated for prophylactic blood patch, particularly if an epidural catheter is in place, they argue that it avoids the need for another epidural puncture, even though strong clinical evidence is lacking [33, 56, 57].

## 11. Summary

Although not life-threatening, PDPH carries substantial morbidity by restricting activities of daily life. Current noninvasive treatments, including bed rest, fluids, analgesics, caffeine, and sumatriptan, only temporize the discomfort [29]. Epidural blood patch remains the invasive treatment of choice, with approximately 70% prolonged success after initial injection [58]. The benefit of prophylactic blood patching is not so clear but deserves consideration in those most at risk from a headache, such as the parturient, and after accidental dural perforation with a Tuohy needle. Surgical closure of the dural tear remains an option of last resort.
